# Patterns and Associated Factors of Congenital Anomalies Among Neonates in 14 Yemeni Governorates 2021–2023: A Case: Control Study

**DOI:** 10.1016/j.gloepi.2025.100196

**Published:** 2025-03-14

**Authors:** Hiam Al-Atnah, Anas Al-Qubati, Amir Addin Al-Hashedi, Muath Al-Saidy, Saleh Al-Shawish, Moamer M. Badi, Najeeb Al-Qubati, Yasser Ghaleb, Maha Al-Muntaser

**Affiliations:** a Faculty of Medicine and Health Sciences, Emirates International University, Sana’a, Yemen; b Ministry of Health and Population, Sana’a, Yemen; c Community Medicine Department, Emirates International University, Sana’a, Yemen

## Abstract

**Background:**

Long-term disability and a reduced quality of life are often associated with congenital anomalies (CAs), which present as structural, functional, or metabolic defects. This study provides a comprehensive view of neonatal congenital anomalies in 14 Yemeni governorates, a significant but often overlooked public health concern. The current study aimed to determine the patterns and associated factors of congenital anomalies in 14 Yemeni governorates between 2021 and 2023.

**Methods:**

An unmatched case-control 1:2 design was conducted using secondary data collected from various health facilities across 14 Yemeni governorates during 2021–2023. Sample size was calculated and data was analyzed using Epi Info version 7.2, with 612 neonates with documented diagnosis of congenital anomalies and 1224 healthy neonates. Binary and multiple logistic regression were used to identify factors associated with congenital anomalies, alongside the chi-square test.

**Results:**

The majority of the congenital anomalies identified were located in Al Hudaydah (34 %), Ibb (17.2 %), and Sana'a (13.1 %). Most were isolated 518 (84.64 %), whereas 94 (15.36 %) were multiple. The predominant system was the nervous system (33.9 %), followed by the skeletal system (14.8 %) and orofacial anomalies (10.6 %). Furthermore, strong associations were found with positive consanguinity (OR = 28.82), low socioeconomic status (OR = 10.70), maternal age ≥ 35 years old (OR = 7.66), stress (OR = 4.95), acute diseases (OR = 3.56), gestational age < 37 weeks (OR = 3.32), maternal age < 20 years old (OR = 2.32), positive family history (OR = 1.74), low birth weight (OR = 1.27), grand-multiparity (OR = 0.71) and male sex (OR = 0.10).

**Interpretation:**

This broad research identified significant patterns, maternal and neonatal associations, and protective variables for congenital anomalies. These results can help inform national interventions and policies for prevention and improving neonatal care.

**Funding:**

This study was self-funded by the authors and did not receive any external funding or any specific grant from funding agencies in the public, commercial, or not-for-profit sectors.

## Introduction

In the vast tableau of human health, congenital anomalies (CAs) are a significant yet often overlooked aspect. CAs are defined as structural, functional, or metabolic defects that can be identified prenatally, at birth, or later in infancy. These anomalies result in long-term impairment and a diminished quality of life. To describe these defects accurately, specific terms are used: malformations, deformations, disruptions, and dysplasias. Malformations are defects in body parts or organs due to intrinsically abnormal development during embryogenesis, typically caused by genetic or environmental factors. In contrast, deformations refer to abnormalities in the position or structure of body parts caused by external mechanical forces, such as uterine constraints, that alter the normal development of a structure. Disruptions are defects that arise when an originally normal development process is interrupted by external factors, such as vascular events or mechanical forces, leading to tissue damage or loss. Dysplasias, on the other hand, involve abnormal cellular organization and growth, resulting in tissues or organs with irregular structure or function. Additionally, CAs can manifest as isolated defects affecting a single organ or system or as part of broader patterns such as syndromes, sequences, field defects, and associations [1,2].

According to severity, CAs are categorized into major and minor anomalies [3]. They can also be divided into three categories: minor, severe, and lethal anomalies. Major anomalies are regarded as both severe and lethal. However, worldwide, CAs are categorized according to the impacted body system [1].

About 25 % of CAs are significantly influenced by genetic factors, 8–12 % due to maternal exposure to environmental toxins (teratogens) that can harm an unborn child, including alcohol, rubella, syphilis, and iodine deficiency, 20–25 % are thought to be multifactorial, and 40–60 % cannot be linked to a specific cause [4].

The prevalence and types of CAs vary by country and even within a country, from area to region. This depends on the definition of CAs adopted, the method of their identification, the length of time, the population under surveillance, and the ethnic and socioeconomic characteristics of the community investigated. Globally, the prevalence rates of CAs range from 2 % to 6 %, and about 8 million neonates are born with CAs annually, with significant variations [5]. The prevalence of CAs is higher in middle-income and low-income countries than in high-income countries [6]. For instance, compared to India, Iran, and Britain, the prevalence of CAs is greater in Egypt, Ethiopia, Kenya, Uganda, and Nigeria [6].

Additionally, CAs significantly contribute to the global burden of diseases. An estimated 295,000 neonates die annually before they reach the age of four weeks due to their congenital disorders and complications and account for 25.3–38.8 million disability-adjusted life years (DALYs), which could be prevented with timely surgery or other interventions [1,2]. Nationally in Yemen, CAs are responsible for roughly >69.9 births per 1000 births and one-fifth of all disabilities, while non-contagious illnesses and injuries account for the remaining 15–16 % of disabilities according to the National Health and Demographic Survey in Yemen conducted in 2013 [7]. Additionally, one out of 10 neonates is born with CAs in a recent study conducted in a tertiary hospital in Sana'a, Yemen, by Thabet SA et al. (2017) [8].

The ongoing cataclysmic war in Yemen, a country on the southern tip of the Arabian Peninsula, has fractured what was once a prosperous country, resulting in the deterioration of the health infrastructure and the unprecedented doubling of CAs, placing neonates in the middle of a dire humanitarian crisis. Taking into account the scarcity of studies on CAs in Yemen and the fact that existing studies are often confined to particular hospitals or governorates to the best of our knowledge, this comprehensive study aims to bridge this critical gap. This study can provide valuable insights specific to Yemen's context and contribute to the cross-cultural worldwide understanding of CAs aiding in the development of both global and national strategies for prevention and management. Considering the immense expenses of treating and rehabilitating children with CAs, our research is not just scientific—it's an imperative step toward a healthier, more equitable world. This study aimed to determine the patterns and associated factors of CAs in 14 Yemeni governorates.

## Methodology

### Study design and study area

The presented study was an unmatched case-control study (1:2). It was carried out by reviewing the clinical files and hospital records of neonates diagnosed with CAs among various hospitals and health centers in 14 Yemeni governorates between 2021 and 2023, namely: Dhamar, Amran, Al Bayda’, Ad Dali’, Sa'dah, Marib, Raymah, Al Hudaydah, Ibb, Sana'a, Hajjah, Al Mahwit, Taizz, and Amanat Al Asimah. We selected the governorates included in this study based on the availability of permission and access, which were limited by the prevailing security situation. These areas were the only ones where we were able to obtain the necessary approvals for data collection. We categorized the data as maternal demographics (maternal age, consanguinity, family history, socioeconomic status, and parity), neonatal demographics (gestational age, sex, weight, and outcome), and associated factors (acute diseases, chronic diseases, drug intake, vaginal bleeding, and proximity to rocket attacks).

### Study population

The study population included neonates diagnosed with CAs in the selected Yemeni governorates between 2021 and 2023. The case group consisted of all neonates diagnosed with CAs from the aforementioned governorates during the study period. To identify the associated factors, 1224 controls were defined as healthy neonates not diagnosed with CAs.

### Sample size and sampling

The sample size was calculated using Epi Info (version 7.2) based on the following parameters which were derived from a similar study (Abdou MSM et al) [9]: a two-sided confidence level of 99.9 %, a power of 90 %, a ratio of controls (group 1) to cases (group 2) of 2:1, a control prevalence of 9 %, an odds ratio of 2, and a case exposure prevalence of 16.5 %. All cases recorded in hospital records were included, while controls were randomly selected from attendees of maternal wards in various hospitals who did not have CAs.

### Data collection

The data was collected using pre-made online Google Sheets for recording information obtained from patients' documents in the archives of the hospitals and facilities. All the study procedures were conducted in a convenient and well-lit room, aided by the facilities' employees. The procedures were regularly evaluated by the team's supervisor for quality assurance. CAs were then classified according to the 11th version of the International Classification of Diseases (ICD-11). We categorized the data as maternal demographics (maternal age, consanguinity, family history, socioeconomic status, and parity), neonatal demographics (gestational age, sex, weight, and outcome), and associated factors (acute diseases, chronic diseases, drug intake, vaginal bleeding, and proximity to rocket attacks). Since income brackets are often difficult to standardize due to ongoing political and economic hurdles such as inflation and currency instability, socioeconomic status was determined via a set of questions. Families categorized as having low socioeconomic status typically lacked stable income, relied on manual labor, informal jobs, or aid, had low educational attainment (e.g., no formal education or primary school only), and lived in poor conditions characterized by crowded housing, inadequate sanitation, and reliance on unsafe water sources. Middle socioeconomic status families had steady but modest income sources, such as small businesses or skilled labor, moderate educational attainment (e.g., high school or some college education), and basic but acceptable living conditions, including access to electricity, clean water, and rented housing. High socioeconomic status families were identified by their higher and stable income sources, such as professionals, business owners, employees in companies or organizations, or expatriates sending remittances, along with higher educational levels (e.g., university degrees) and superior living conditions, including owned housing, insured healthcare, and access to various amenities.

### Statistical analysis

Data was digitized and analyzed using Epi Info version 7.2. Frequencies and percentages were calculated to describe the common types of CAs. Univariate analysis with the chi-square test or Fisher's exact test, as appropriate were used in no expected cell count less than 1 and at most 20 % of expected cell counts less than 5 to assess the differences in demographic characteristics of cases and controls. Crude odds ratios (cORs) and adjusted odds ratios (aORs) with 95 % confidence intervals (CIs) were calculated to measure the strength and significance of the association between CAs and maternal and neonatal demographics and associated factors. All variables obtained by binary analysis were included in the multivariate logistic regression to identify factors independently associated with CAs. Each factor was coded, and those considered a risk were coded as 1 and those not considered a risk were coded as 0. Similarly, in factors with multiple categories, we considered one group as a reference and the others as risks.

### Ethical approval

The present study obtained ethical approval from Emirates International University and the Yemeni Ministry of Health and Population to gain access to all hospitals and health centers' management, staff, and archives. They were informed that participation is voluntary and that they can refuse this without stating any reason.

### Data availability

The authors had full access to all the data and final responsibility for the decision to submit for publication. The data underlying this study cannot be shared publicly due to patient confidentiality regulations. However, we are happy to share the analytical methods that were employed upon requesting the corresponding author.

### Role of funding

This study was self-funded by the authors and did not receive any specific grant from funding agencies in the public, commercial, or not-for-profit sectors. The authors had full access to all the data in the study and shared responsibility for the decision to submit for publication.

## Results

**Fig. 1:**
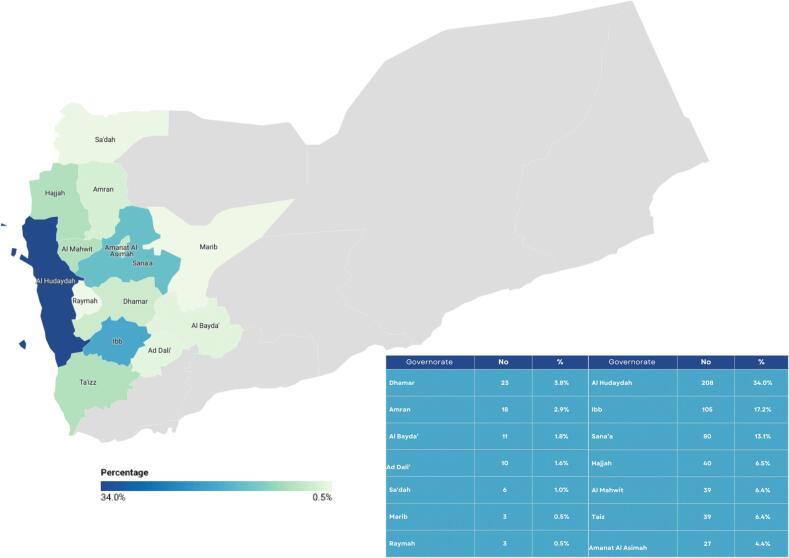
Distribution of CA cases among governorates. (Created with Datawrapper and Canva).

In this study, CAs patterns were analyzed using an unmatched 1:2 ratio case-control approach. The study population consisted of 612 cases and 1224 controls. The highest CAs rates were in Al Hudaydah (34 %), Ibb (17.2 %), and Sana'a (13.1 %). Whereas Sa'dah (1 %), Marib (0.5 %), and Raymah (0.5 %) had low rates of CAs. (Fig. 1).

**Fig. 2:**
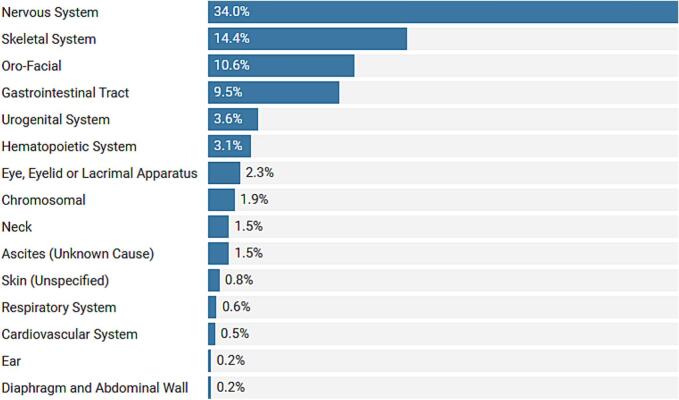
Distribution of CA cases according to body system. (Created with Datawrapper).

The majority of the CAs were identified as isolated 518 (84.6 %), whereas 94 (15.4 %) were multiple—two or more anomalies on a single case involving two or more systems. The highest proportion of CAs were those of the nervous system (208, 33.9 %), followed by the skeletal system (88, 14.8 %) and oro-facial anomalies (65, 10.6 %). Further details of the distribution of CAs are shown in Fig. 2.

The most common nervous system anomalies were hydrocephalus (13.8 %) and anencephaly (13.0 %), followed by spina bifida (2.7 %). The most common skeletal anomalies were unspecified limb anomalies (6 %), amelia (4.2 %), and clubfoot (2.4 %). While the most common oro-facial anomalies were cleft lip (7.8 %) and cleft lip and palate (1.4 %). Further details are shown in Table 1.

**Table 1:** Distribution of CA cases according to ICD-11 classification.

Body System	ICD -11 Code	Anomaly	No	%
Nervous System	LA04	Congenital Hydrocephalus	85	13.8
	LA00	Anencephaly	80	13.1
	LA02	Spina Bifida	17	2.7
	LA01	Encephalocele	11	1.7
	LA0Y	Unspecified	10	1.6
	LA05.0	Microcephaly	3	0.4
	LA03	Arnol-Chiari Malformation	2	0.3
Multiple Anomalies	LD2Z	Unspecified	94	15.3
Skeletal System	LB9Z	Limbs Anomalies	37	6.0
	LB9A.0-LB99.0	Amelia	26	4.2
	LB98.00-LB98.22	Club foot	15	2.4
	LB78	Polydactyly	6	0.9
	LB79	Syndactyly	4	0.6
Oro-Facial Anomalies	LA40	Cleft Lip	48	7.8
	LA4Y	Cleft Lip and Palate	9	1.4
	LA5Z	Facial Anomalies	6	0.9
	LA42	Cleft Palate	2	0.3
Gastrointestinal Tract Anomalies	LB15.*Z*-LB16.Z	Intestinal CAs	17	2.7
	LB01	Omphalocele	16	2.6
	LB02	Gastroschisis	10	1.6
	LB17.0	Imperforated Anus	5	0.8
	DB50.2	Congenital Anorectal Fistula	3	0.4
	LB16.0	Intestinal Atresia	3	0.4
	LB16.1	Hirschsprung's Disease	2	0.3
	LB12.1	Esophageal Atresia	2	0.3
Urogenital System	LB5Z	Genital Anomalies (Male)	12	1.9 %
	LB31.0	Hydronephrosis	6	0.9 %
	LB4Z	Genital Anomalies (Female)	3	0.4 %
	GB8Y	Polycystic Kidney	1	0.1 %
Hematopoietic System	KA85.Z	Hydrops Fetalis	19	3.1
Eye	LA10.1	Anophthalmia	8	1.3 %
	9C61	Congenital Glaucoma	3	0.4 %
	LA1Z	Unspecified	3	0.4 %
Chromosomal Anomalies	LD40.0	Down Syndrome	7	1.1 %
	LD40.1	Patau Syndrome	3	0.4 %
	LD40.2	Edward Syndrome	2	0.3 %
Neck	LA6Z	Neck Anomalies	7	1.1 %
	LA60	Webbed neck	2	0.3 %
Ascites (Unknown Cause)	ME04.Z		9	1.4 %
Skin	LC7Z	Unspecified	5	0.8 %
Respiratory System	KB2B	Lung Immaturity	5	0.8 %
Circulatory System	LA8Z	Congenital Heart Disease	3	0.4 %
Ear	LA2Z	Unspecified	1	0.1 %
Diaphragm and Abdominal Wall	LB00.0	Diaphragmatic Hernia	1	0.1 %

In terms of maternal demographics, older mothers (≥35 years) (OR = 9.76, 95 % CI = 7.77–12.27) and neonates born to parents with positive consanguinity (OR = 32.90, 95 % CI = 24.22–44.73) were more likely to develop CAs. Other factors significantly associated with higher occurrence of CAs included low socioeconomic status (OR = 26.41, 95 % CI = 13.82–50.48), positive family history (OR = 4.29, 95 % CI = 3.27–5.62), and grand multiparity (≥5 births) (OR = 3.51, 95 % CI = 2.48–4.96), and. Conversely, high socioeconomic status and nulliparity or multiparity were protective factors. Further details are shown in Table 2.

**Table 2:** Maternal demographics, neonatal demographics, and associated factors among CA cases and unmatched controls.

Variable	Category	Studied Group *(n* *=* *612)*		Controlled Group *(n* *=* *1224)*		OR	95 % CI	
		No	%	No	%		Lower	Upper
*Maternal Demographics*								
Maternal Age	Mean (SD)	29.7 ± (6.8)						
	<20	22	3.5 %	48	3.9 %	2.32	1.37	3.93
	20- < 35 *(Ref)*	192	31.3 %	970	79.2 %			
	≥35	398	65 %	206	16.8 %	9.76	7.77	12.27
Consanguinity	Yes	385	62.9 %	60	4.9 %	32.90	24.22	44.73
	No	227	37 %	1164	95.1 %			
Family History	Yes	169	27.6 %	100	8.1 %	4.29	3.27	5.62
	No	443	72.3 %	1124	91.8 %			
Socioeconomic Status	Low	496	81 %	400	32.6 %	26.41	13.82	50.48
	Middle	106	17.3 %	611	49.9 %	3.70	1.90	7.20
	High *(Ref)*	10	1.6 %	213	17.4 %	–	–	–
Parity	Nullipara *(Ref)*	61	9.9 %	161	13.1 %	–	–	–
	Multipara	280	45.7 %	859	70.1 %	0.86	0.62	1.19
	Grandpara	271	44.2 %	204	16.6 %	3.51	2.48	4.96
*Neonatal Demographics*								
Gestational Age	Mean ± (SD) = 36.16 ± 4.37							
	<37 weeks	373	60.9 %	356	29 %	4.41	2.25	8.64
	37–42 weeks	217	35.5 %	724	59.1 %	1.25	0.64	2.46
	≥42 weeks *(Reference)*	22	3.6 %	144	11.6 %	–	–	–
Sex	Male	478	78.1 %	525	42.8 %	4.75	3.80	5.93
	Female	134	21.9 %	699	57.1 %			
Neonatal Demographics								
Fetal Weight: Mean ± (SD) = 2854.20 ± 555								
	<2500 g	373	60.9 %	356	29 %	4.41	5.09	10.56
	2500- < 3500 g	217	35.5 %	724	59.1 %	1.25	0.87	1.65
	≥3500 g *(Reference)*	22	3.6 %	144	11.6 %	–	–	–
Outcome	Alive	321	52.4 %	1199	97.9 %	0.02	0.02	0.04
	Stillbirth	291	47.5 %	25	2.04 %			
*Associated Factors*								
Acute Diseases	Yes	53	8.6 %	45	3.6 %	2.48	1.65	3.74
	No	559	91.3 %	1179	96.3 %			
Chronic Disease	Yes	32	5.2 %	18	1.4 %	3.70	2.06	6.64
	No	580	94.7 %	1206	98.5 %			
Drugs	Yes	145	23.6 %	127	10.3 %	2.68	2.06	3.48
	No	467	76.3 %	1097	89.6 %			
Vaginal Bleeding	Yes	40	6.5 %	36	2.9 %	2.30	1.46	3.66
	No	572	93.5 %	1188	97.1 %			
Stress	Yes	97	15.8 %	36	2.9 %	6.22	4.18	9.24
	No	515	84.1 %	1188	97.0 %			
Proximity to Rocket Attacks	Yes	86	14.0 %	43	3.5 %	4.49	3.07	6.57
	No	526	85.95 %	1181	96.4 %			

In the analysis of neonatal demographics, the study indicated a significant association between gestational age, gender, birth weight, and CAs. Preterm neonates (<37 weeks) (OR = 4.41, 95 % CI = 2.25–8.64) and low-birth-weight neonates (<2500 g) (OR = 4.41, 95 % CI = 5.09–10.56) had higher risks of CAs. Male neonates also demonstrated higher odds (OR = 4.75, 95 % CI = 3.80–5.93). Additionally, neonates with CAs also had a higher risk of stillbirth and reduced survival at birth. Conversely, neonates born between 37 and 42 weeks, post-term neonates (≥42 weeks), those with a birth weight of ≥2500 g, and female neonates were associated with lower odds of CAs.

Furthermore, we identified associations between multiple maternal variables during pregnancy and the development of CAs. Acute diseases during pregnancy (OR = 2.48, 95 % CI = 1.65–3.74), chronic diseases (OR = 3.70, 95 % CI = 2.06–6.64), drug intake (OR = 2.68, 95 % CI = 2.06–3.48), stress (OR = 6.22, 95 % CI = 4.18–9.24), and proximity to rocket attacks (OR = 4.49, 95 % CI = 3.07–6.57) were all related with an increased risk of anomalies (Table 4.5).

All significant variables that were obtained by binary analysis were included in multiple logistic regression analysis (Table 3), which was conducted for 13 variables, namely acute disease, chronic disease, consanguinity, stress, vaginal bleeding, drugs, gestational age, maternal age, parity, fetal weight, sex, family history, and socioeconomic status. Ten variables were identified that significantly affected the occurrence of CAs: consanguinity (OR = 28.82), low socioeconomic status (OR = 10.70), maternal age (OR = 7.66), stress (OR = 4.95), acute diseases (OR = 3.56), gestational age (OR = 3.32), family history (OR = 1.74), birth weight (OR = 1.27), parity (OR = 0.71), and sex (OR = 0.10).

**Table 3:** Logistic regression analysis of associated factors with CA cases.

Term	OR	95 %	C·I
Consanguinity (Yes/No)	28.82	18.62	44.6
Socioeconomic Status	10.70	7.34	15.61
Maternal Age	7.66	5.07	11.58
Stress (Yes/No)	4.95	2.55	9.61
Acute Disease (Yes/No)	3.56	1.74	7.29
Gestational Age	3.32	2.35	4.71
Family History (Yes/No)	1.74	1.07	2.82
Weight	1.27	0.76	2.11
Parity	0.71	0.46	1.10
Sex	0.10	0.07	0.15

## Discussion

This study investigated key variables associated with CAs in 14 Yemeni governorates. In Yemen, where the prevalence of CAs is on the rise, our study highlights its singularity as a broader and more comprehensive study on the patterns and associated factors of CAs and one of the few existing studies on CAs in general. All maternal demographics, neonatal demographics, and associated factors identified were notably associated with the occurrence of CAs. However, the major variables identified after logistic regression were consanguinity, low socioeconomic status, maternal age ≥ 35 years old, and maternal stress.

In terms of the patterns and distribution of CAs, the majority were isolated, and a minority were multiple. The nervous system had the highest proportion of CAs, followed by the musculoskeletal system and oro-facial abnormalities. These results align with studies from South India [10], Ethiopia [11], and Baghdad, Iraq [12], which also found that the nervous system and the skeletal systems were the most predominant. On the contrary, a retrospective analytic study conducted in Aden, Yemen [13] showed the digestive system, the cardiovascular system, and the urogenital system as the most predominant alongside the nervous and skeletal systems. Another retrospective study at a tertiary care hospital showed the digestive, nervous, and musculoskeletal systems as the most frequent [8]. This discrepancy could be attributed to the fact that anomalies involving the digestive, cardiovascular, and urogenital systems were present in conjugation with other anomalies, classifying them as multiple anomalies in our study. Furthermore, in this study, only a minority of neonates were diagnosed with cardiac anomalies when further specialized investigations, such as echocardiography, were warranted after clinical examination, and the majority were referred to specialized cardiac centers for documented diagnosis. This highlights the need for more thorough diagnostic procedures in neonatal care and comprehensive documentation follow-up for neonates referred to specialized centers.

This study found a strong link between consanguinity and the probability of developing new CAs. This is similar to studies conducted in Saudi Arabia and Erbil, Iraq [14,15]. Consanguineous marriages are popular in various parts of the Middle East, Africa, and the Indian subcontinent [16], with one estimate claiming that “one billion people live in communities with a preference for consanguineous marriage” (Hamy, 2012) [17]. This predilection has significant societal roots. Nonetheless, education, combined with preconception and premarital counseling, can be effective preventative techniques by raising awareness and enabling couples to make more educated decisions.

Furthermore, mothers ≥35 years old were more likely to give birth to neonates with CAs in addition to those <20 years old. Many studies concerning the risk factors of CAs showed associations between increasing maternal age and incidence of CAs [18,19]. This also aligns with similar studies conducted in Baghdad, Iraq [20] and Alexandria, Egypt [9] where mothers ≥35 were at a higher risk of having CAs. This could be owing to the risk-increasing effect of chromosomal aberrations, which become more common with advanced maternal age. Hollier et al. propose that the accumulation of environmental exposures over time may also increase the risk [21]. Additionally, studies conducted in Mosul, Iraq [22] and Ethiopia [23] found that younger mothers have a higher risk, which were attributed to nuclear waste and the effect of environmental factors, such as teratogenic drug intake during early pregnancy, respectively. Particular attention should be devoted to more frequent discrepancies in maternal age groups. Examining what may be driving each age group's risk-increasing effects will help find the best preventative approaches. The teratogenic effects associated with mothers becoming pregnant at a young age, as well as the lack of primary prevention options, may largely explain this age group's vulnerability, including smoking and drug intake, lower social status, lower educational attainment, and a lack of adequate folic acid supplementation [24,25].

Additionally, mothers of low socioeconomic status were at a higher risk of developing CAs, while those of higher class were at a lower risk. This is congruent to the study conducted in Baghdad, Iraq [26], where low socioeconomic status affects the health of the mother and the fetus via inadequate nutrition and poor obstetric follow-up. The burden of CAs is evidently higher in developing countries, especially among the low socioeconomic groups [27]. This is because developing countries, including Yemen, face various obstacles in combating CAs, including limited access to healthcare facilities, insufficient antenatal care, and environmental variables that contribute to congenital disability. These obstacles are aggravated by social and economic inequality, where individuals and families frequently face poverty, poor education, and a lack of resources [28]. This multifaceted interplay of factors highlights the grave need for comprehensive interventions to mitigate the incidence of CAs in these vulnerable populations.

Furthermore, stress was a significant associated factor. This is supported by a population-based case-control study from 1987 to 1989, which indicated that having at least one stressful occurrence during the periconceptional period was connected to a prevalence odds ratio of 1.4–1.5 for the delivery of neonates with conotruncal heart abnormalities, neural tube anomalies, and isolated cleft lip with or without palate. According to this study, women who endured stressful life events around conception or early gestation were more likely to give birth to infants with certain congenital abnormalities [29]. In another study, pregnant women experiencing positive events during pregnancy had a lower risk of CHD in offspring than those without positive events (OR = 0.38, 95 %, CI: 0.30–0.48). The risk of CHD in offspring could increase by 62 % among the pregnant women experiencing the negative events compared to those without (OR = 1.62, 95 %, CI: 1.29–2.03) [30].

## Limitation

The main limitations of this study included recall bias. Additionally, further research is needed to cover the remaining Yemeni governorates and further delve into specific associated factors to determine their causality and relations to the timing of exposure. Moreover, unmeasured confounding remains a limitation, as not all potential confounding factors could be accounted for in the analysis. For example, the absence of genetic testing data restricts the ability to explore genetic factors comprehensively, since a quarter of CAs are caused by genetic defects.

## Conclusion

In conclusion, this study bridged a critical gap in Yemen regarding CAs and utilized all abundant but fragmented statistics, reports, and records across 14 Yemeni governorates. Since published national referencing studies were limited to hospitals in a single governorate, this resulted in a limited insight into the patterns and associated factors of these anomalies and highlighted the need for broader, more comprehensive study. Further research is needed to cover the remaining Yemeni governorates and further delve into specific associated factors to determine their causality and relations to the timing of exposure. Additionally, employing a digital monitoring system and the creation of a national registry will enhance data collection, standardize reporting, and facilitate better tracking of CAs over time. Also, investigating the factors contributing to the high prevalence of CAs in governorates such as Al-Hudaydah, Ibb, and Sana'a, alongside legally mandating premarital counseling, will address critical gaps in prevention efforts. This will help healthcare providers make informed decisions regarding the planning of pregnancy, optimizing antenatal care, and mitigating the burden of CAs in Yemen.

## Contributors

**Authors:**

• Hiam Al-Atnah and Anas Al-Qubati: Literature review, final data quality revision, and writing of the research report and manuscript.
• Amir Addin Al-Hashedi, Muath Al-Saidy, Saleh Al-Shawish: Data collection supervision, data quality review, and research report drafting.
• Yasser Ghaleb: Data Analysis and Manuscript Review.
• Maha Al-Montasser: Review, editing and proofreading of the research report and manuscript.
• Najeb Al-Qubati: Data Access Permission Acquisition.
• Moamer M.Badi: General Supervision and review of the research report and manuscript.

**Data Collectors:**

• Mohammed Damaj: Initial proposal draft writing and data collection.
• Saleh Albasarah, Jamal Jameel, Abdullah Goruon, Abdulrahman Al-Thawr, Wissam Albaseer: Data collection.

## CRediT authorship contribution statement

**Hiam Al-Atnah:** Writing – review & editing, Writing – original draft, Visualization, Validation, Resources, Project administration, Methodology, Investigation, Data curation, Conceptualization. **Anas Al-Qubati:** Writing – review & editing, Writing – original draft, Visualization, Validation, Resources, Project administration, Methodology, Investigation, Data curation, Conceptualization. **Amir Addin Al-Hashedi:** Writing – original draft, Visualization, Validation, Data curation. **Muath Al-Saidy:** Writing – original draft, Visualization, Validation, Data curation. **Saleh Al-Shawish:** Writing – original draft, Visualization, Validation, Data curation. **Moamer M. Badi:** Writing – review & editing, Visualization, Validation, Supervision, Methodology, Data curation. **Najeeb Al-Qubati:** Writing – review & editing, Validation, Resources, Data curation, Conceptualization. **Yasser Ghaleb:** Writing – review & editing, Methodology, Formal analysis. **Maha Al-Muntaser:** Writing – review & editing, Writing – original draft, Validation.

## Declaration of competing interest

The authors declare that they have no known competing financial interests or personal relationships that could have appeared to influence the work reported in this paper.
